# A Rare Case of Diltiazem-Induced Photosensitivity

**DOI:** 10.7759/cureus.40376

**Published:** 2023-06-13

**Authors:** Maria A Perozo, Lorena Escaño, Rachel Thompson, Harold Cedeno

**Affiliations:** 1 Internal Medicine, Danbury Hospital, Danbury, USA; 2 Internal Medicine, University of Vermont, Vermont, USA

**Keywords:** diltiazem, calcium channel blockers, allergic skin reactions, sun-exposed area, skin reaction, medication side-effects, photosensitivity reaction

## Abstract

Photosensitivity is a condition of heightened skin sensitivity to sunlight or other forms of ultraviolet light. The case presented here highlights a rare occurrence of diltiazem-induced photosensitivity in an 87-year-old female patient with a history of atrial fibrillation and multiple comorbidities. The patient developed a distinctive erythematous rash limited to the sun-exposed area of the left side of her face during her hospital stay for respiratory failure and pneumonia. The localized distribution of the rash, along with its resolution upon reducing sun exposure, strongly suggested a photosensitivity reaction induced by diltiazem. Prompt discontinuation of the medication and transitioning to verapamil led to the resolution of the cutaneous manifestations. This case emphasizes the importance of recognizing and managing photosensitivity reactions as potential adverse effects of medications, such as diltiazem. Further research is needed to better understand the pathophysiology and risk factors associated with photosensitivity reactions to calcium channel blockers. Through this case report, we aim to contribute to the existing knowledge in this field and emphasize the significance of vigilance in clinical practice.

## Introduction

Photosensitivity is a condition in which the skin becomes very sensitive to sunlight or other forms of ultraviolet light and may burn easily. Photosensitivity usually causes a rash or sunburn, especially on areas of the skin that are exposed to the ultraviolet light. The affected areas may be painful and may itch, blister, or peel. Photosensitivity may be caused by certain medications, such as antibiotics and anticancer drugs, radiation therapy, exposure to certain chemicals, and some medical conditions, such as lupus and xeroderma pigmentosum [1,2].

Diltiazem is a benzothiazepine calcium channel blocker; it is widely prescribed for the treatment of hypertension and angina. While its most common side effects include edema, headache, and dizziness, it is important to note that diltiazem can also lead to various cutaneous side effects. Adverse skin reactions associated with calcium channel blockers are generally infrequent and primarily manifest as pruritus, rashes, and urticaria. In the literature, there has been a limited number of reports documenting photosensitive reactions specifically linked to nifedipine and diltiazem. In rare instances, severe reactions such as Stevens-Johnson syndrome, erythema multiforme, and photosensitive lichen planus have been reported. Although photodistributed hyperpigmentation cases have been more extensively documented, the incidence of photosensitivity reactions remains relatively scarce [1-7].

## Case presentation

Here, we present a case of an 87-year-old Caucasian female with a significant medical history, including recently diagnosed atrial fibrillation with rapid ventricular response (RVR), for which she was initiated on diltiazem. She also had a history of chronic obstructive pulmonary disease (COPD), diabetes mellitus, hyperlipidemia, chronic kidney disease, major depressive disorder, and generalized anxiety disorder. The patient had no previous history of allergic reactions to any medications.

The patient initially presented to the hospital complaining of shortness of breath and cough productive of clear sputum. On arrival at the emergency department, she was in acute respiratory distress. She was afebrile, tachycardic, tachypneic, and normotensive, with oxygen saturation of 88% on room air. Supplemental oxygen was administered via nasal cannula at a rate of 3 L. Laboratory tests revealed mild leukocytosis with neutrophilic predominance and increased lactic acid levels. Chest X-ray showed a hazy opacity at the left lung base, suggestive of a new infiltrate. Electrocardiogram confirmed atrial fibrillation with RVR.

**Figure 1:**
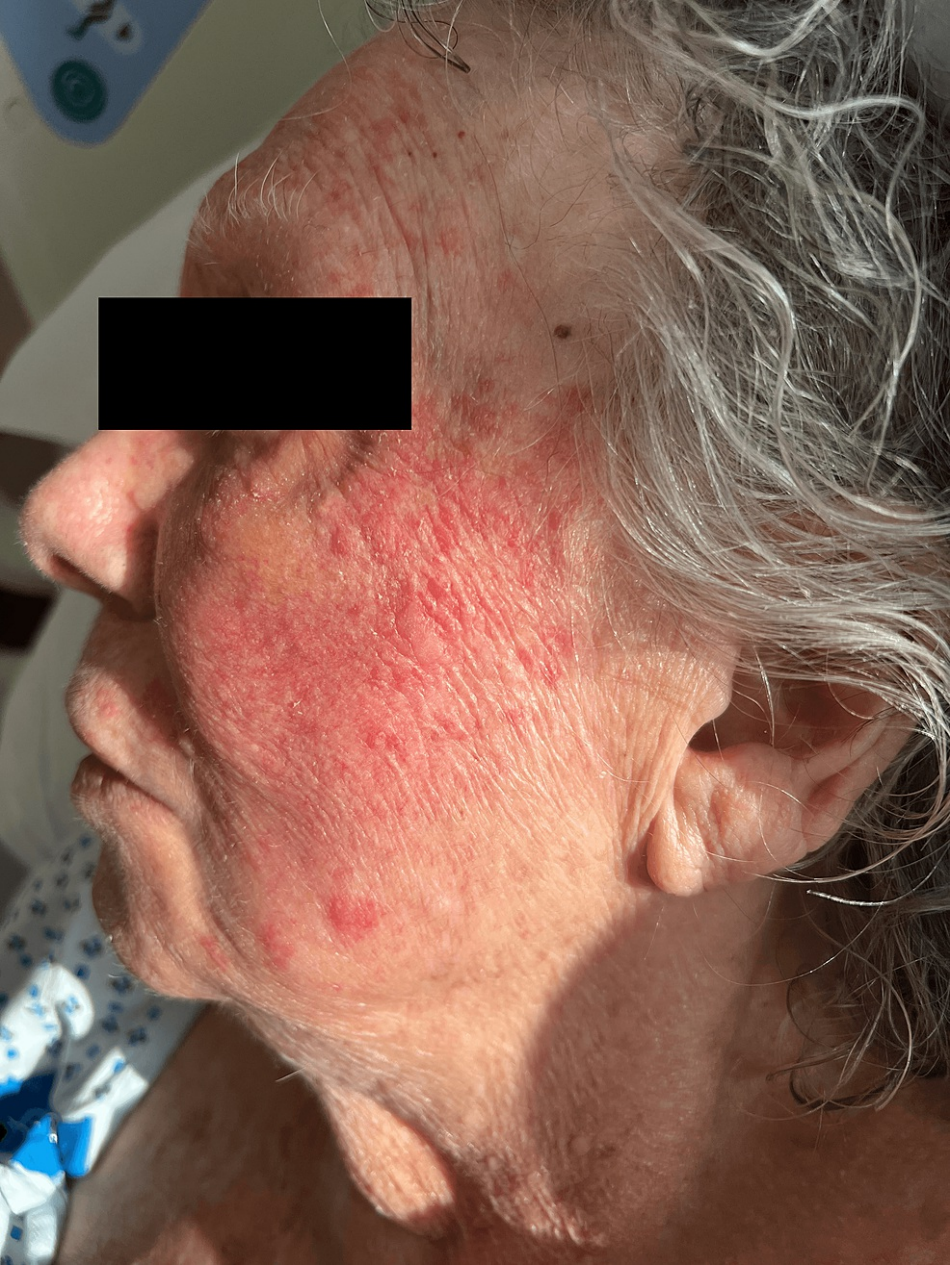
Image showing the rash limited to the sun-exposed area on the left side of the patient's face, with sun exposure in the early morning

The patient was admitted to the hospital for further management of acute hypoxemic respiratory failure secondary to COPD exacerbation and pneumonia. Treatment was initiated with ceftriaxone, azithromycin, prednisone taper, and inhalers as per protocol. Her home medications were continued. On the sixth day of hospitalization, the patient developed a cutaneous eruption consisting of a reticular erythematous blanching rash on the left side of her face. Notably, the rash was limited to the area exposed to ultraviolet light coming through the windows (Figure 1). Physical examination did not reveal any cutaneous findings in other parts of the body. Precautions were taken to cover the windows with curtains. The patient denied associated symptoms such as pain, pruritus, fever/chills, or difficulty breathing. She also denied previous similar rashes and reported limited sun exposure prior to admission. Her vital signs and laboratory results were within normal limits.

**Figure 2:**
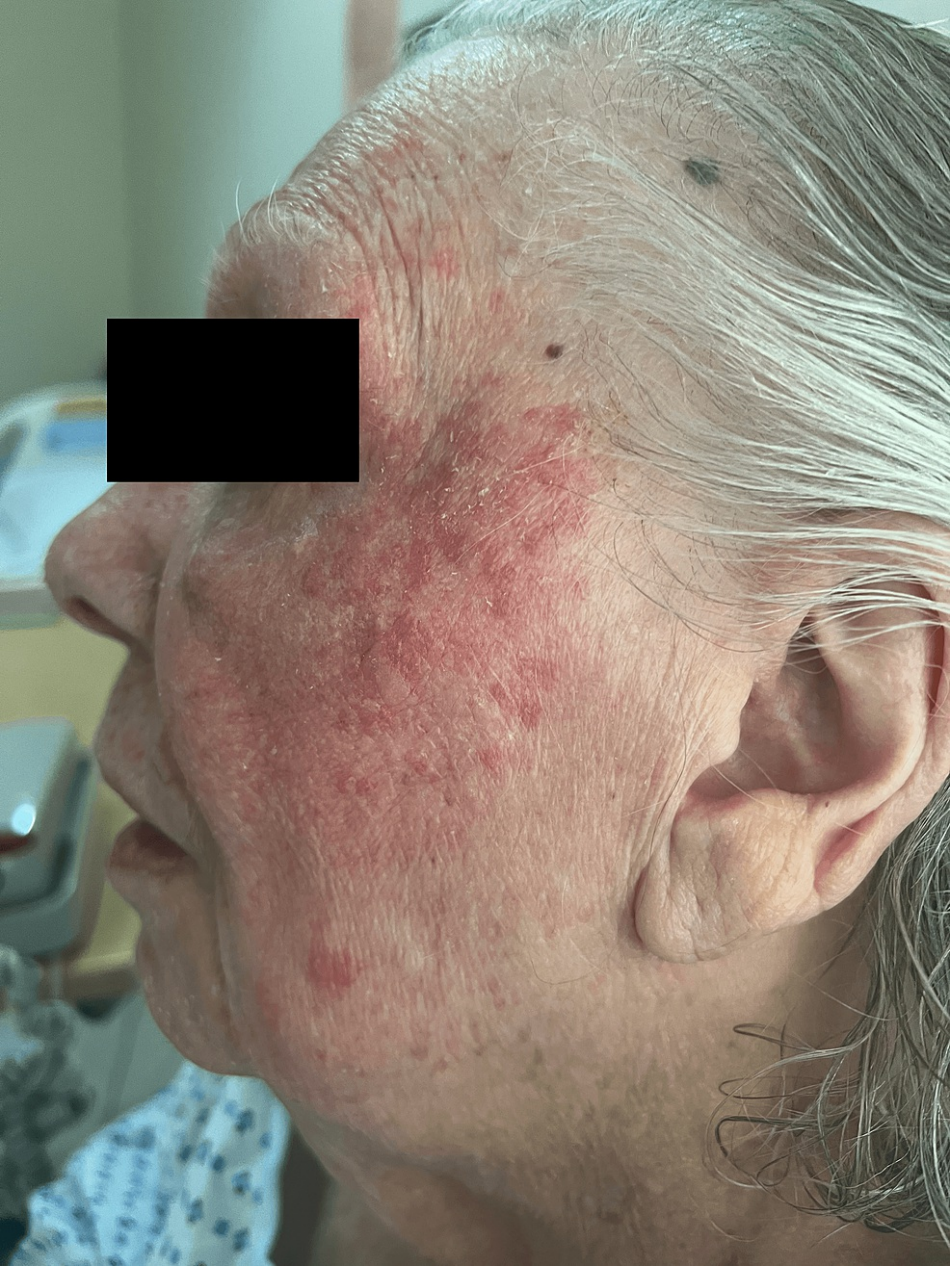
Improvement in the rash two hours after sun exposure was minimized The patient had complete resolution of symptoms afterwards.

Given the new-onset rash limited to the sun-exposed areas, medication-induced photosensitivity dermatitis was suspected. The list of medications was reviewed, and diltiazem was identified as the only medication associated with photosensitivity dermatitis. Importantly, diltiazem had been initiated three weeks prior to the development of the rash. Consequently, the decision was made to discontinue diltiazem and transition the patient to verapamil. Sun exposure was also minimized, leading to the subsequent resolution of the cutaneous findings (Figure 2, showing improvement two hours after sun exposure was minimized).

Following this admission, the patient has been readmitted to the hospital for her chronic lung problems. No further episodes of photosensitive cutaneous eruptions have been reported since the discontinuation of diltiazem and reduction of sun exposure.

## Discussion

We have presented here a compelling case report involving an 87-year-old female patient who was initially hospitalized for acute hypoxic respiratory failure related to pneumonia. During her previous hospitalization, she was diagnosed with newly discovered atrial fibrillation with rapid ventricular response and started on metoprolol. However, due to significant wheezing observed in the current hospitalization, metoprolol was switched to diltiazem at a dosage of 30 mg every six hours.

An intriguing aspect of this case was the emergence of a distinctive erythematous rash exclusively on the sun-exposed area of the patient's left side of the face during her hospital stay. The subsequent improvement of the rash upon reducing sun exposure raised suspicions of photosensitivity as the potential underlying cause. Consequently, diltiazem was promptly discontinued and the medication was transitioned to verapamil.

The clinical course of our patient, coupled with the localized distribution of the skin lesions, strongly supports the diagnosis of a photosensitivity reaction specifically induced by diltiazem. The resolution of the rash following the discontinuation of the medication further reinforces this conclusion. Since there is a suitable alternative, a drug challenge was not performed. Notably, the exact pathogenic mechanism underlying this reaction remains elusive. It is worth noting that despite the widespread use of calcium channel blockers, the reported incidence of cutaneous adverse reactions is relatively low, with non-specific rashes being more commonly reported in the literature [1].

## Conclusions

In summary, our case emphasizes the critical importance of recognizing and considering photosensitivity reactions as potential adverse effects of medications, including diltiazem. Prompt identification and management, such as discontinuing the offending drug and minimizing sun exposure, play a pivotal role in achieving the resolution of cutaneous manifestations. Further research is warranted to gain a deeper understanding of the pathophysiology and identify risk factors associated with photosensitivity reactions to calcium channel blockers. By shedding light on this intriguing case, we contribute to the growing body of knowledge in this field and underscore the significance of vigilance in clinical practice.
